# Characterization of myocardial remodeling with diffusion tensor magnetic resonance imaging in chronic porcine model using the toroid-based representation

**DOI:** 10.1186/1532-429X-11-S1-P79

**Published:** 2009-01-28

**Authors:** Choukri Mekkaoui, Marcel P Jackowski, Donald P Dione, Francis G Spinale, Albert J Sinusas

**Affiliations:** 1 Yale University, New Haven, CT USA; 2 University of São Paulo, São Paulo, Brazil; 3 Medical University of South Carolina, Charleston, SC USA

**Keywords:** Infarct Region, Myocardial Remodel, Myocardial Fiber, Fiber Angle, Diffusion Tensor Magnetic Resonance Image

## Introduction

Diffusion Tensor Magnetic Resonance Imaging (DT-MRI) is a non-invasive technique capable of characterizing myocardial fiber architecture and structural properties [1], and may provide new insights about remodeling after myocardial infarction (MI). The helical organization and anisotropic nature of myofibers require efficient strategies for visualization and analysis. In this work, a toroid-based model of the diffusion tensor is used to create an improved depiction of myofiber orientation and derive a new diffusivity map, the toroidal volume (TV). This is applied to characterize regional tissue structure and fiber angle distribution in normal and post-MI remodeled porcine hearts.

## Purpose

To evaluate the structure and fiber organization of normal porcine hearts and hearts 2- and 8-weeks post-MI using TV maps derived from the toroid-based representation of DT-MRI collected on a 3 T MR scanner.

## Methods

LV remodeling was assed in 5 normal porcine hearts and hearts 2- and 8-weeks post surgical ligation of marginal branches of LCX coronary artery. Animals were euthanized and hearts were carefully excised and perfused with saline solution. Each heart was then placed in a container and filled with Fomblin (Ausimont, Thorofare, NJ). DT-MRI was performed on a 3.0 T scanner (Siemens, Erlangen, Germany) using a segmented EPI sequence, 6 gradient directions; b-values = 0 (T2-weighted) and 600 s/mm^2^; voxel-size = 2 × 2 × 2 mm^3^; 50 short-axis slices; TR = 5400 ms; TE = 84 ms; 40 averages (EPI factor = 7). Based on T2-weighted images, tissue was classified into Infarct (I), Non-infarct (NI), and Border (B) regions. The toroid-based representation of the DT is described by the modified parametric equation of an elliptical torus and TV is the volume of the toroid. Fiber angle variance (FAV) captures the degree of fiber angles spreading over the volume of interest. TV (mm^6^/s^3^) and FAV (deg.^2^) indices were then quantified for 16 segments of the standard cardiac polar map.

## Results

**Figure 1:**
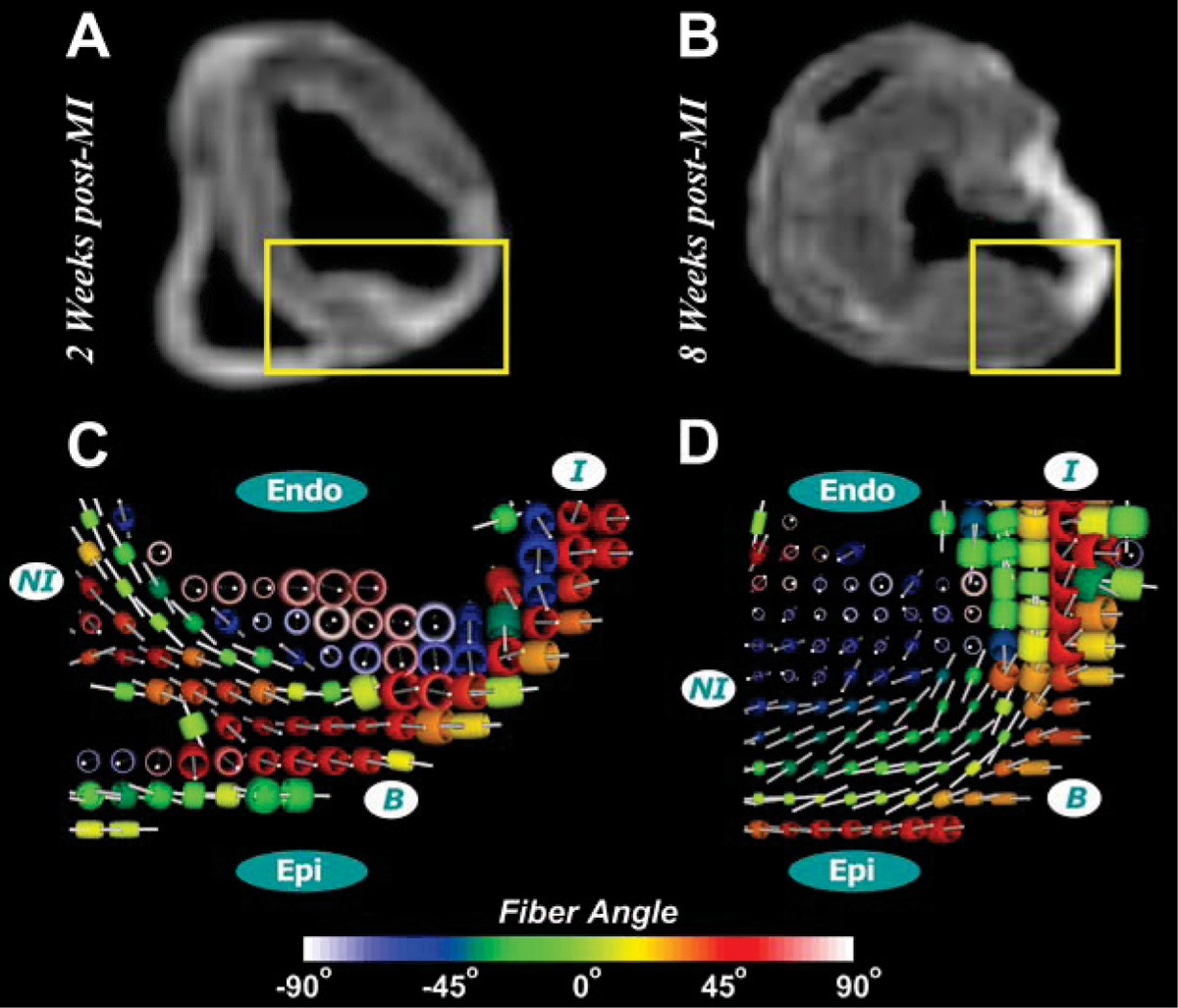
**(A) and (B) mid-ventricular T2-weighted image of a 2- and 8-weeks post-MI pig hearts, respectively**. The yellow rectangles represent the ROIs used for toroidal glyph visualization, which includes ***NI, I*** and ***B*** regions. Glyph are colored according to the fiber inclination angle as defined by Scollan *et al.* (1998, *Am J Physiol*). (**C**) Myocardial fiber angles in the infarct region at 2 weeks post-MI change from helical to more longitudinal with reorganization at 8 weeks post-MI (less longitudinal) (**D**).

**Figure 2:**
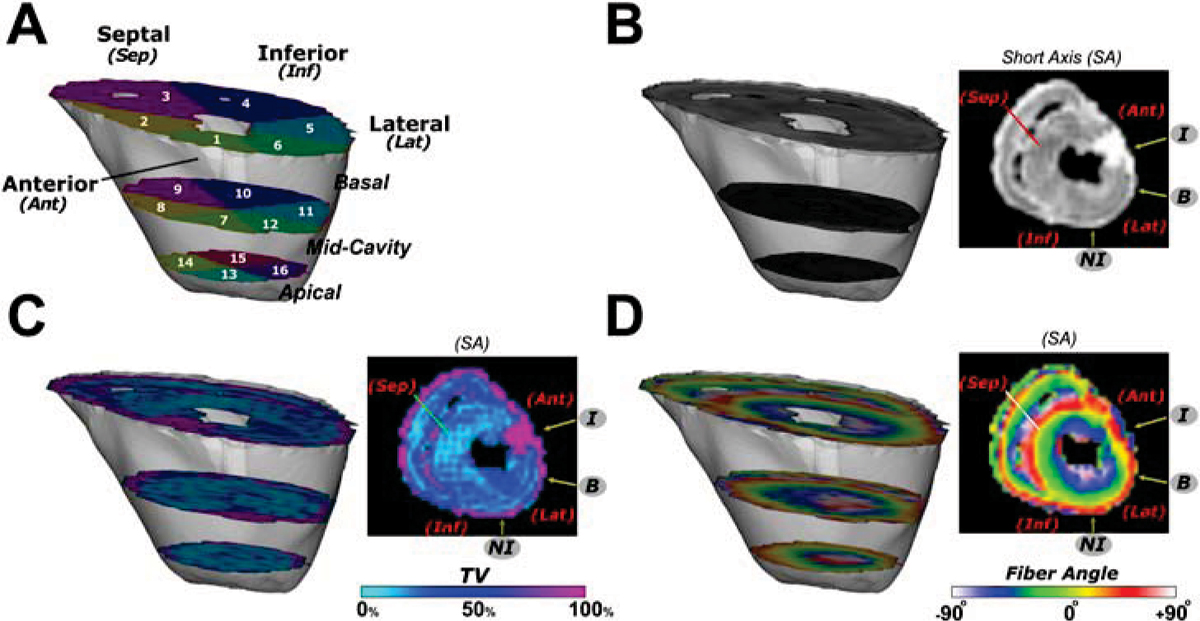
**A three-dimensional view of an 8-weeks post-MI heart, with the myocardial segmentation is shown (A)**. The other frames show a mid-ventricular slice of a T2-weighted image (**B**), a color-coded map of TV expressed as a percentage (**C**) and the color-coded fiber inclination angle map (**D**). The **I** nfarct and **B** order regions are clearly distinguishable in the TV map and the laminar fiber architecture is altered in the **I** nfarct region.

**Figure 3:**
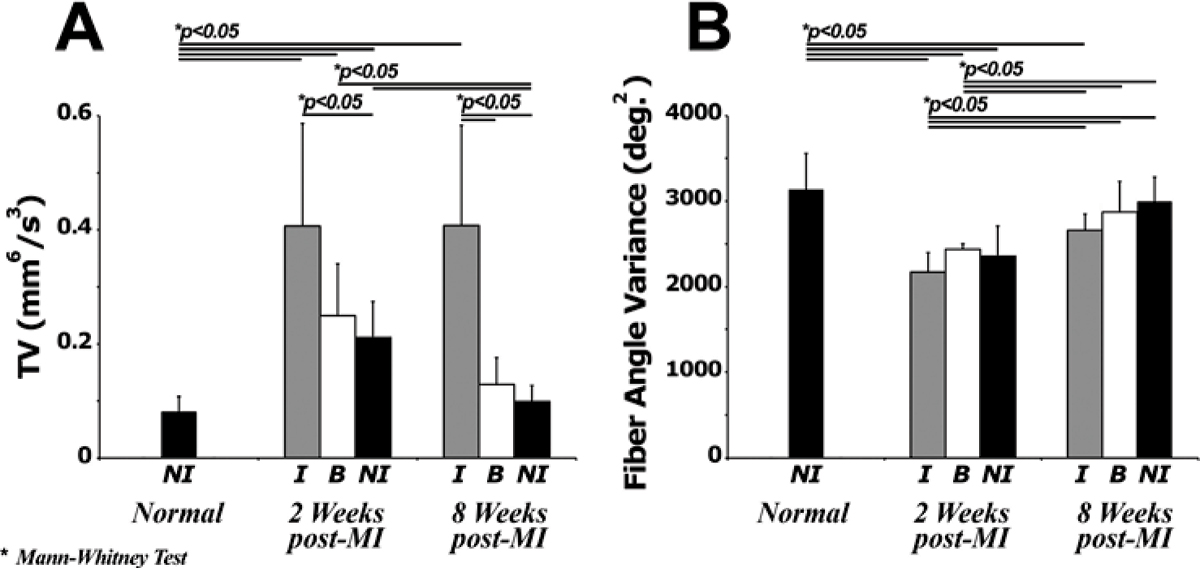
**Mean TV (A) and fiber angle variance (B) computed from 16 regions of the standard cardiac polar map for normal, 2- and 8-weeks post-MI hearts**. Myocardial regions were classified as ***NI, B*** or ***I*** based on the T2-weighted image. TV and fiber angle variance for all categories is significantly elevated at 2 weeks post-MI and remains elevated only in the infarcted region at 8 weeks post-MI. These findings indicate the progress of the remodelling process post-MI.

The toroidal representation revealed that myocardial fiber angles in the infarct region at 2-weeks post-MI changed from helical to more uniform longitudinal orientation, with reorganization at 8-weeks post-MI (less longitudinal) (Figure 1). This toroid-based approach exhibits the laminar architecture with the changing orientation of fiber angles from epicardium to endocardium and also reveals a change in the toroidal shape in the infarct region. The structural change is reflected by a substantial global increase in TV. Hence, the analysis of TV and FAV maps allows for a structural and a geometrical quantification of the myocardial remodeling (see Figure 2 for an example at 8-weeks post-MI). TV and FAV were uniform in normal hearts, and significantly (p < 0.05) altered in the infarct regions at 2- and 8-weeks post-MI. TV and FAV were also altered (p < 0.05) in NI and B regions at 2-weeks post-MI with normalization by 8-weeks (Figure 3). These changes in TV and FAV are in agreement with previously observed spatial and temporal changes in regional activation of matrix metalloproteinase's [2].

## Conclusion

Results suggest that the DT-MRI indices of TV and FAV provide quantitative information that may enhance the understanding of the underlying myocardial structure properties and temporal changes involved in the post-MI remodeling process.
